# Endothelium-protective, histone-neutralizing properties of the polyanionic agent defibrotide

**DOI:** 10.1172/jci.insight.149149

**Published:** 2021-09-08

**Authors:** Hui Shi, Alex A. Gandhi, Stephanie A. Smith, Qiuyu Wang, Diane Chiang, Srilakshmi Yalavarthi, Ramadan A. Ali, Chao Liu, Gautam Sule, Pei-Suen Tsou, Yu Zuo, Yogendra Kanthi, Evan A. Farkash, Jiandie D. Lin, James H. Morrissey, Jason S. Knight

**Affiliations:** 1 Division of Rheumatology, Department of Internal Medicine, University of Michigan, Ann Arbor, Michigan, USA.; 2 Division of Rheumatology, Ruijin Hospital, Shanghai Jiao Tong University School of Medicine, Shanghai, China.; 3 Department of Biological Chemistry, University of Michigan, Ann Arbor, Michigan, USA.; 4 Life Sciences Institute and Department of Cell & Developmental Biology, University of Michigan Medical Center, Ann Arbor, Michigan, USA.; 5 Division of Intramural Research National Heart, Lung and Blood Institute Bethesda, Maryland, USA.; 6 Division of Cardiovascular Medicine, Department of Internal Medicine and; 7 Department of Pathology, University of Michigan, Ann Arbor, Michigan, USA.

**Keywords:** Inflammation, Vascular Biology, Endothelial cells, Neutrophils, Thrombosis

## Abstract

Neutrophil-mediated activation and injury of the endothelium play roles in the pathogenesis of diverse disease states ranging from autoimmunity to cancer to COVID-19. Neutralization of cationic proteins (such as neutrophil extracellular trap–derived [NET-derived] histones) with polyanionic compounds has been suggested as a potential strategy for protecting the endothelium from such insults. Here, we report that the US Food and Drug Administration–approved polyanionic agent defibrotide (a pleiotropic mixture of oligonucleotides) directly engages histones and thereby blocks their pathological effects on endothelium. In vitro, defibrotide counteracted endothelial cell activation and pyroptosis-mediated cell death, whether triggered by purified NETs or recombinant histone H4. In vivo, defibrotide stabilized the endothelium and protected against histone-accelerated inferior vena cava thrombosis in mice. Mechanistically, defibrotide demonstrated direct and tight binding to histone H4 as detected by both electrophoretic mobility shift assay and surface plasmon resonance. Taken together, these data provide insights into the potential role of polyanionic compounds in protecting the endothelium from thromboinflammation with potential implications for myriad NET- and histone-accelerated disease states.

## Introduction

Neutrophils are the most abundant innate effector cells of the human immune system, exerting antimicrobial effects through phagocytosis and degranulation (1). The release of neutrophil extracellular traps (NETs) — web-like structures composed of microbicidal cytosolic and granule proteins enmeshed in decondensed chromatin — is a more recently described strategy by which neutrophils kill microbes in tissues (2). However, when formed intravascularly, NETs are potentially noxious, trapping RBCs, activating platelets, and damaging the endothelium — thereby promoting coagulation, vascular occlusion, and thrombosis (3–6). Endothelial activation and injury driven by NETs has been revealed as a key pathogenic step in a variety of disease states, including deep vein thrombosis (7), transfusion-related acute lung injury (8), atherosclerosis (9), and lupus (10). High levels of NETs have also been detected in the blood of coronavirus disease 2019 (COVID-19) patients (11), where they likely contribute to the endothelial damage regularly noted on the histopathology of COVID-19 organs (12–15).

NETs present a variety of highly cationic proteins, including histones, HMGB1, calprotectin, cathepsin G, and LL-37, among others. While these proteins contribute to the capture and inactivation of invading microorganisms, they may also be cytotoxic to host tissues (16). In particular, NET-derived histones account for approximately 70% of NET-associated proteins (16) and have been associated with endothelial damage and multiple-organ dysfunction in acute states such as sepsis (17), acute pancreatitis (18), acute respiratory distress syndrome (19), and severe trauma (20). High levels of circulating histones (up to 250 μg/mL after trauma; ref. 20) activate and damage endothelial cells via pore formation (21, 22); engagement of innate sensors, such as TLRs (23–25) and the NLRP3 inflammasome (26, 27); and forced release of von Willebrand factor (vWF; ref. 28). The result is a hypercoagulable state and an increased risk of vascular events including thrombosis. Targeting histones by neutralizing their cationic nature with polyanions has been suggested as an approach to combatting various NET- and histone-associated diseases (29, 30).

Defibrotide is a pleotropic mixture of oligonucleotides (90% single-stranded and 10% double-stranded phosphodiester oligonucleotides) that is derived from porcine intestinal mucosal DNA and that has antithrombotic, fibrinolytic, and antiinflammatory activities (31, 32). Defibrotide was initially approved for the treatment of thrombophlebitis and as prophylaxis for deep vein thrombosis in Italy (refs. 33, 34; note that these approvals are no longer active). Subsequently, it was granted an orphan drug designation by European and American regulatory agencies for the treatment of serious hepatic veno-occlusive disease (VOD) after hematopoietic cell transplantation (Europe) or VOD with renal and/or pulmonary dysfunction after transplant (United States) (34). Although defibrotide’s mechanisms of action remain incompletely understood, there is evidence that it protects endothelium, modulates platelet activation, potentiates fibrinolysis, decreases thrombin generation and activity, and reduces circulating levels of plasminogen activator inhibitor type 1 (35–39). Defibrotide has also been demonstrated to associate with cationic proteins — for example, collagen I (40).

Here, we hypothesized that the polyanionic properties of defibrotide might mitigate activation of and damage to the endothelium by NETs and especially NET-derived cationic proteins. In pursuit of this possibility, we characterized defibrotide’s endothelium-protective properties both in vitro and in a mouse model of venous thrombosis.

## Results

### Defibrotide inhibits the activation of cultured endothelial cells by NETs.

**Figure 1:**
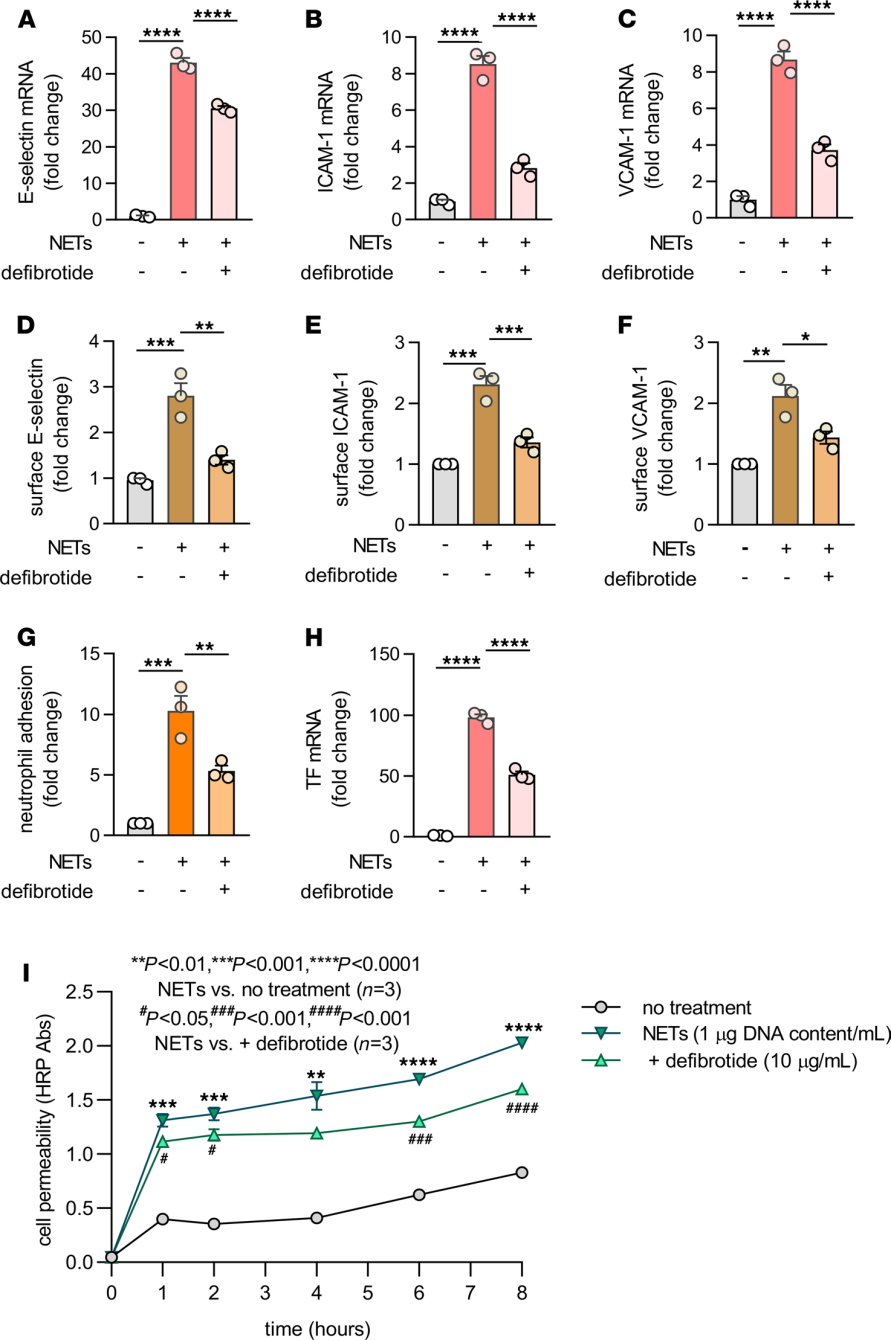
Defibrotide inhibits the activation and permeability of cultured HUVECs by NETs. (**A**–**C**) HUVECs were pretreated with defibrotide (10 μg/mL) for 30 minutes, followed by isolated NETs (1 μg DNA content/mL) for 4 hours. E-selectin (**A**), ICAM-1 (**B**), and VCAM-1 (**C**) mRNA levels were determined by qPCR. Mean ± SD is presented for 1 representative experiment out of 3 independent experiments, all with similar results; ****P < 0.0001 by 1-way ANOVA corrected by Dunnett’s test. (**D**–**F**) HUVECs were pretreated with defibrotide (10 μg/mL) for 30 minutes, followed by the addition of NETs for 6 hours. Surface expression of E-selectin (**D**), ICAM-1 (**E**), and VCAM-1 (**F**) were then detected by in-cell ELISA. (**G**) HUVEC monolayers were pretreated with defibrotide (10 μg/mL) for 30 minutes, followed by NETs (1 μg DNA content/mL) for 4 hours. Calcein-AM–labeled neutrophils were then added as described in Methods. Mean ± SD is presented for *n =* 3 independent experiments; **P < 0.01 and ***P < 0.001 by 1-way ANOVA corrected by Dunnett’s test. (**H**) HUVECs were treated as for **A**–**C**. Tissue factor mRNA levels were detected at 4 hours. Mean ± SD is presented for 1 representative experiment out of 3 independent experiments, all with similar results; ****P < 0.0001 as compared by 1-way ANOVA corrected by Dunnett’s test. (**I**) HUVECs were treated as for **A**–**C**. Cell permeability was assessed by measuring horseradish peroxidase (HRP) movement through EC monolayers in a Transwell system as described in Methods. Mean ± SD is presented for 1 representative experiment out of 3 independent experiments, all with similar results; **P < 0.01, ***P < 0.001 and ****P < 0.0001 by 2-way ANOVA corrected by Tukey’s test. ^#^*P <* 0.05, ^###^*P <* 0.001, and ^####^*P <* 0.0001 by 2-way ANOVA corrected by Tukey’s test.

Human umbilical vein endothelial cells (HUVECs) were cultured with human neutrophil–derived NETs in the presence or absence of defibrotide. Gene transcripts associated with the expression of cell adhesion molecules E-selectin, ICAM-1, and VCAM-1 were then quantified. In all cases, expression was markedly increased by purified NETs, whether those NETs were originally triggered by phorbol 12-myristate 13-acetate (PMA) (Figure 1, A–C) or calcium ionophore (Supplemental Figure 1). These NET-mediated increases were consistently restrained in the presence of defibrotide (Figure 1, A–C, and Supplemental Figure 1; supplemental material available online with this article; https://doi.org/10.1172/jci.insight.149149DS1). Beyond gene expression, we also confirmed that purified NETs increased surface protein expression of E-selectin, ICAM-1, and VCAM-1 via an in-cell ELISA assay, and we confirmed that these increases could be mitigated by defibrotide (Figure 1, D–F). We reasoned that if these expression differences were functionally meaningful, then adhesion of neutrophils to the HUVEC monolayer should track in a similar fashion (increased by NETs and decreased by defibrotide). As predicted, calcein-AM–labeled human neutrophils adhered more strongly to NET-activated HUVECs, an effect that was reduced in the presence of defibrotide (Figure 1G). Beyond surface adhesion molecules, previous work has also suggested that NETs upregulate expression of tissue factor (TF) by endothelial cells, thereby contributing to the prothrombotic state (41). Here, we found that TF was upregulated by NETs whether measured by gene expression (Figure 1H) or enzymatic activity (Supplemental Figure 2); in both contexts, NET-mediated increases were significantly reduced by defibrotide (Figure 1H and Supplemental Figure 2). Finally, we examined the effect of defibrotide on NET-regulated permeability of HUVEC monolayers. Indeed, NETs increased permeability across HUVEC monolayers in as little as 1 hour, whereas the addition of defibrotide reduced this NET-mediated increase (Figure 1I). Taken together, these data support the basic premise of the study, namely that defibrotide can neutralize the activation and permeability of endothelial cells by NETs.

### Transcriptome profiling confirms a NET-induced proinflammatory signature in endothelial cells, which can be mitigated by defibrotide.

**Figure 2:**
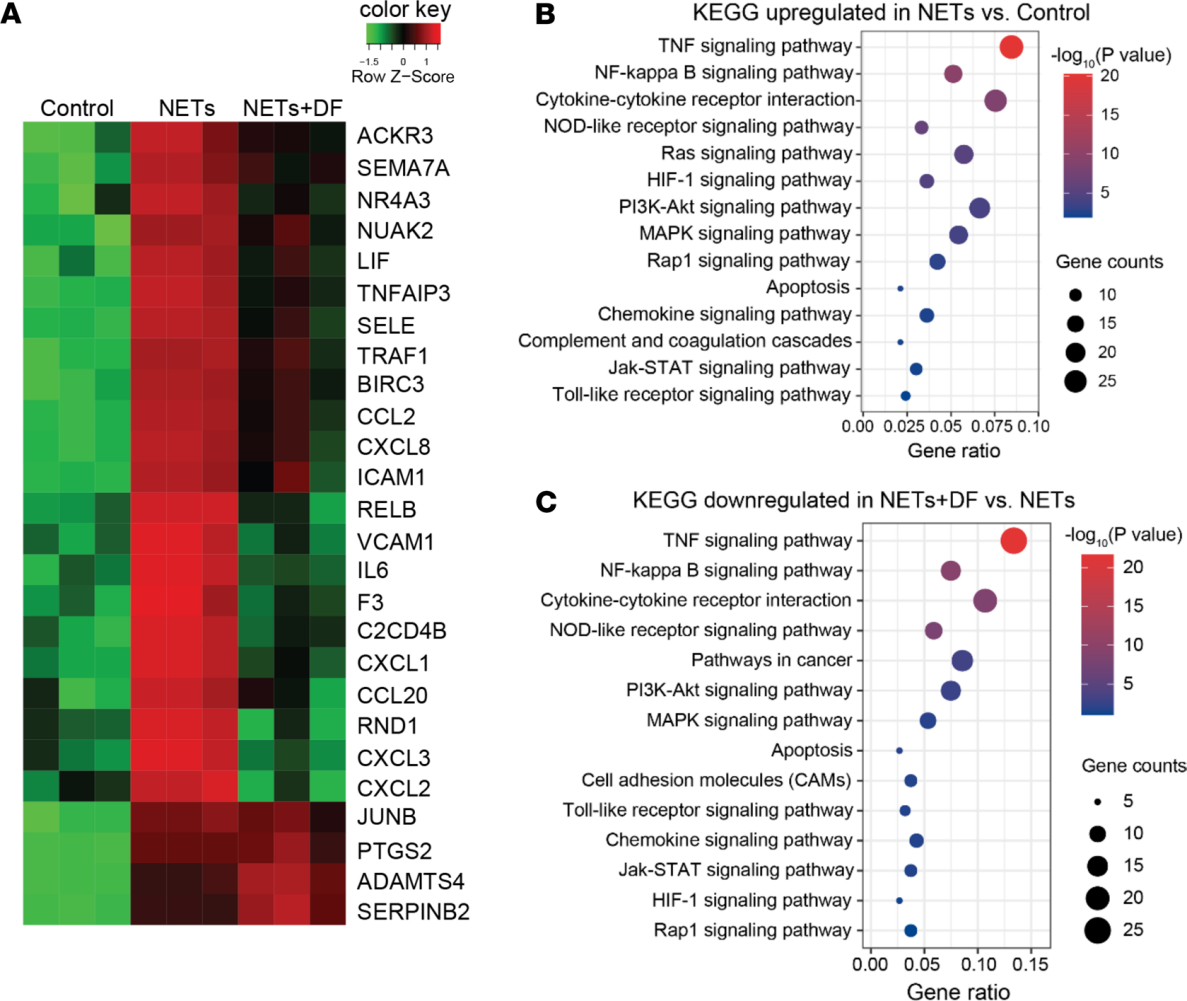
Transcriptome profiling of HUVECs in response to NETs ± defibrotide. (**A**) HUVECs were treated with vehicle (PBS), NETs (1 μg DNA content/mL), or NETs + defibrotide (10 μg/mL) for 4 hours (*n =* 3 per group). RNA sequencing was performed. K-means clustering of differentially expressed genes is presented as a heatmap. (**B**) Bubble plot of upregulated biological processes in the NETs group as compared with the vehicle group. Color-coding is based on *P* value, and bubble size is based on the number of genes in each pathway. (**C**) Bubble plot of downregulated biological processes in the NETs group as compared with the NETs + defibrotide group. DF, defibrotide.

The above data demonstrate activation of endothelial cells by NETs in the context of selected genes associated with cell-cell interactions and coagulation. To more broadly understand the pathways associated with endothelial cell activation, we performed RNA sequencing (RNA-seq) of HUVECs exposed to vehicle, NETs, or NETs with defibrotide (NETs + defibrotide). We identified 440 differentially expressed genes (300 upregulated) in HUVECs upon NET stimulation as compared with vehicle. Conversely, there were 229 differentially expressed genes (192 downregulated) when the NETs + defibrotide group was compared with NETs alone. The top upregulated genes are displayed in Figure 2A. Functional gene network analysis of upregulated genes in NET-stimulated HUVECs revealed an inflammatory signature highlighted by meta groups such as the TNF signaling pathway, NF-κB signaling pathway, and MAPK signaling pathway (Figure 2B). Notably, the same pathways that were upregulated by NETs were likely to be downregulated by defibrotide (Figure 2C). Taken together, these data confirm the ability of NETs to activate endothelial cells, and they demonstrate the ability of defibrotide to reverse those effects.

### Blocking histone H4 counteracts HUVEC activation by NETs.

**Figure 3:**
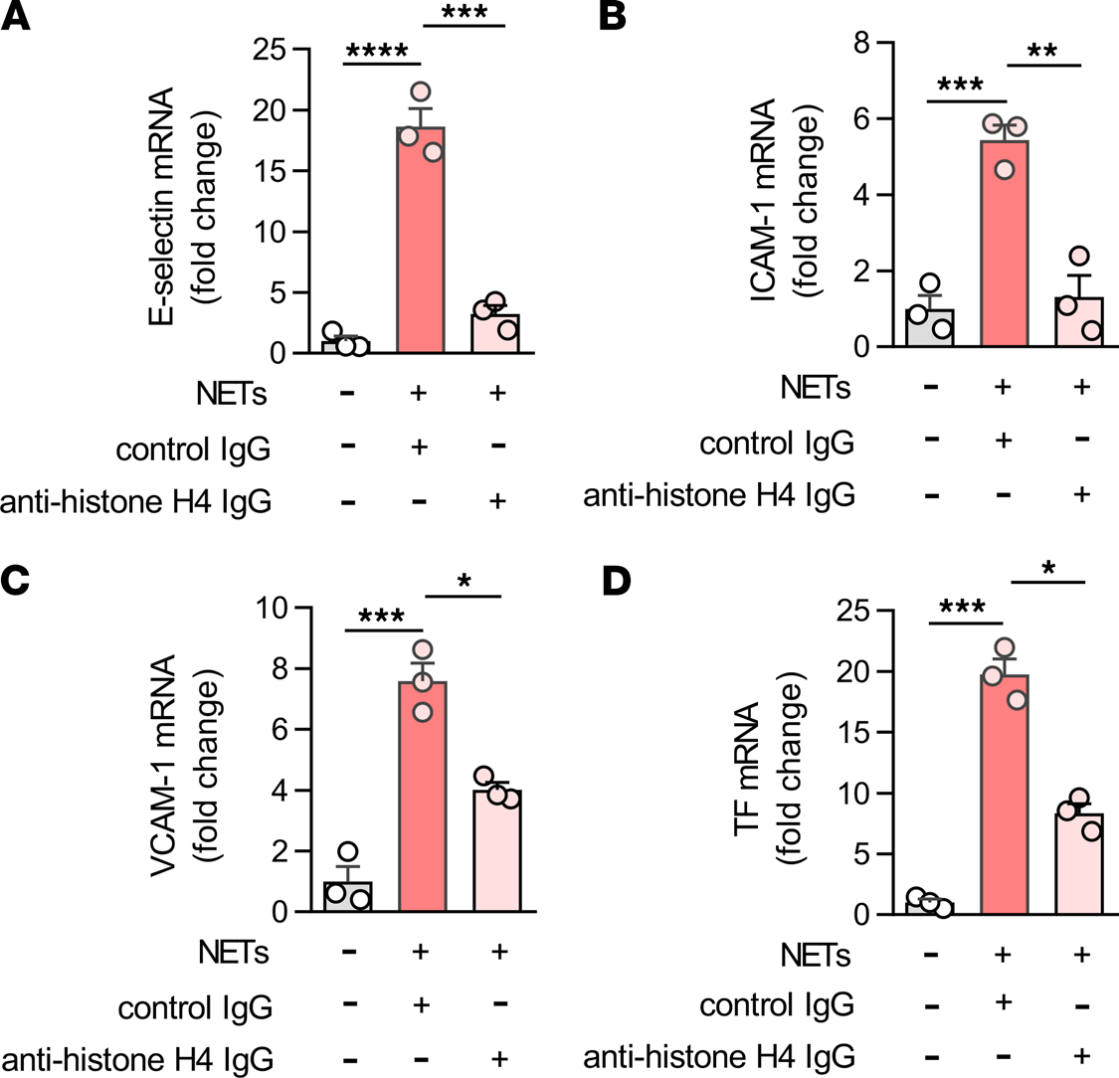
NET-derived histone H4 induces HUVEC activation. (**A**–**D**) NETs (1 μg DNA content/mL) were incubated with antibodies to histone H4 (100 ng/mL) for 1 hour and then added to HUVECs for 4 hours. E-selectin (**A**), ICAM-1 (**B**), VCAM-1 (**C**), and tissue factor (TF) mRNA levels were determined by qPCR. Mean ± SD is presented for 1 representative experiment out of 3 independent experiments, all with similar results; *P < 0.05, **P < 0.01, ***P < 0.001,and ****P < 0.0001 by 1-way ANOVA corrected by Dunnett’s multiple comparison test.

As discussed above, part of the original hypothesis was that the polyanionic nature of defibrotide might make it especially effective at neutralizing NET-derived cationic proteins such as histones (2), which are important mediators of inflammation, tissue injury, and organ dysfunction in the extracellular space (42, 43). To begin to address this, we asked whether a histone-neutralizing antibody might be effective in our system. Indeed, an anti–histone H4 antibody counteracted the upregulation of HUVEC E-selectin (Figure 3A), ICAM-1 (Figure 3B), VCAM-1 (Figure 3C), and TF (Figure 3D) by NETs.

### Defibrotide abolishes endothelial cell activation by extracellular histone H4.

**Figure 4:**
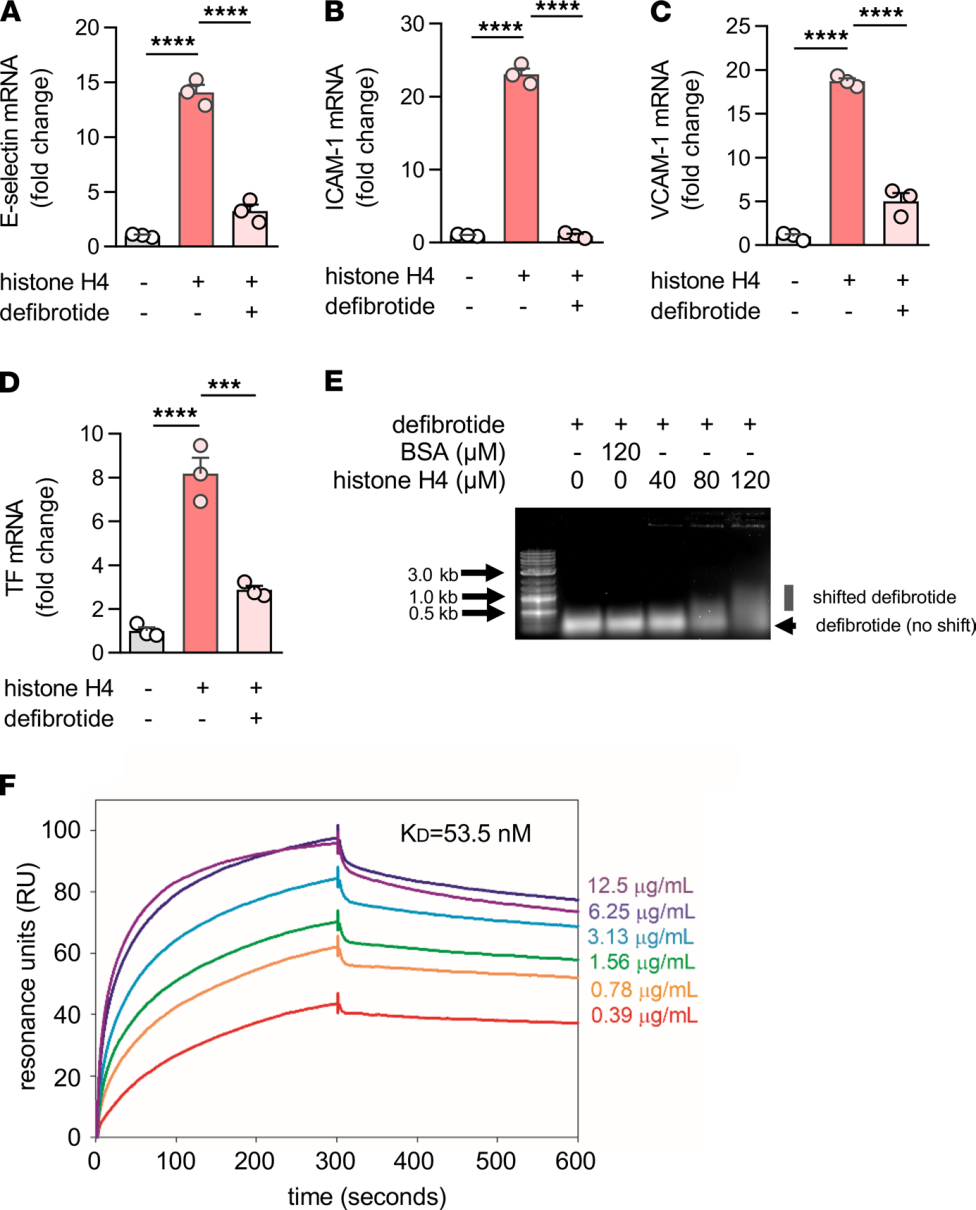
Defibrotide abolishes HUVEC activation by extracellular histone H4. (**A**–**D**) HUVECs were pretreated with defibrotide (10 μg/mL) for 30 minutes, followed by recombinant histone H4 (25 μg/mL) for 4 hours. E-selectin (**A**), ICAM-1 (**B**), VCAM-1 (**C**), and tissue factor (TF) (**D**) mRNA levels were determined by qPCR. Mean ± SD is presented for 1 representative experiment out of 3 independent experiments, all with similar results; ***P < 0.001, ****P < 0.0001 by 1-way ANOVA corrected by Dunnett’s test. (**E**) Defibrotide, and histone H4 were incubated at 37°C for 30 minutes and then resolved on a 0.5% agarose gel. (**F**) Surface plasmon resonance assay characterizing the binding kinetics of defibrotide to histone H4. The profile of defibrotide at gradient concentrations (from 0.39 μg/mL to 12.5 μg/mL) flowing over histone H4 protein immobilized on a NiNTA chip are shown. The calculated dissociation constant (*K_D_*) is labeled.

We next asked whether defibrotide might directly antagonize the effects of histone H4. As expected, purified histone H4 increased expression of E-selectin (Figure 4A), ICAM-1 (Figure 4B), and VCAM-1 (Figure 4C) by HUVECs, while defibrotide almost completely abolished these effects. Given that citrullinated histones are important components of NETs, we also treated HUVECs with citrullinated histone H4. We found similar activation as for native histone H4 and again found that the effect was significantly restrained by defibrotide (Supplemental Figure 3); native histone H4 was, therefore, used in all subsequent experiments. Beyond HUVECs, we questioned whether defibrotide could also protect microvascular endothelial cells against histone H4. We treated human dermal microvascular endothelial cells (HDMVECs) with histone H4 and defibrotide, and we found a similar pattern as for HUVECs. Specifically, gene transcripts for E-selectin, ICAM-1, VCAM-1, and TF were upregulated by histone H4 and were then restrained in the additional presence of defibrotide (Supplemental Figure 4). Mechanistically, we found that both TLR2 and TLR4 were involved in histone H4–mediated endothelial cell activation (Supplemental Figure 5) as has been previously reported (44). Inflammatory cytokines (IL-8 and MCP-1) also increased in HUVEC supernatants upon exposure to histone H4 and were subsequently suppressed by defibrotide (Supplemental Figure 6). Similar patterns were also observed for TF gene expression (Figure 4D) and enzymatic activity (Supplemental Figure 7). Given these findings, along with the RNA-seq data, we investigated whether defibrotide might be working to counterbalance intracellular signaling pathways associated with TLRs or TNF signaling. However, defibrotide showed only mild protection when HUVECs were activated by TNF-α and little to no protection when HUVECs were activated by LPS (Supplemental Figure 8). We therefore next tested whether defibrotide might work through direct engagement with histone H4 in the extracellular space. Indeed, using an electrophoretic mobility shift assay (EMSA; ref. 45), we found evidence of a direct interaction between histone H4 and defibrotide in that histone H4 could slow the migration of defibrotide (a mixture of oligonucleotides) through an agarose gel (Figure 4E). In contrast, histone H4 had no impact on the migration of BSA. A strong interaction between histone H4 and defibrotide was also confirmed by surface plasmon resonance (SPR). Assuming an average molecular weight of defibrotide as 16.5 kD, the equilibrium dissociation constant (*K_D_*) between defibrotide and histone H4 was calculated as 53.5 nM (Figure 4F).

### Defibrotide strongly protects endothelial cells against histone H4–induced cell death.

**Figure 5:**
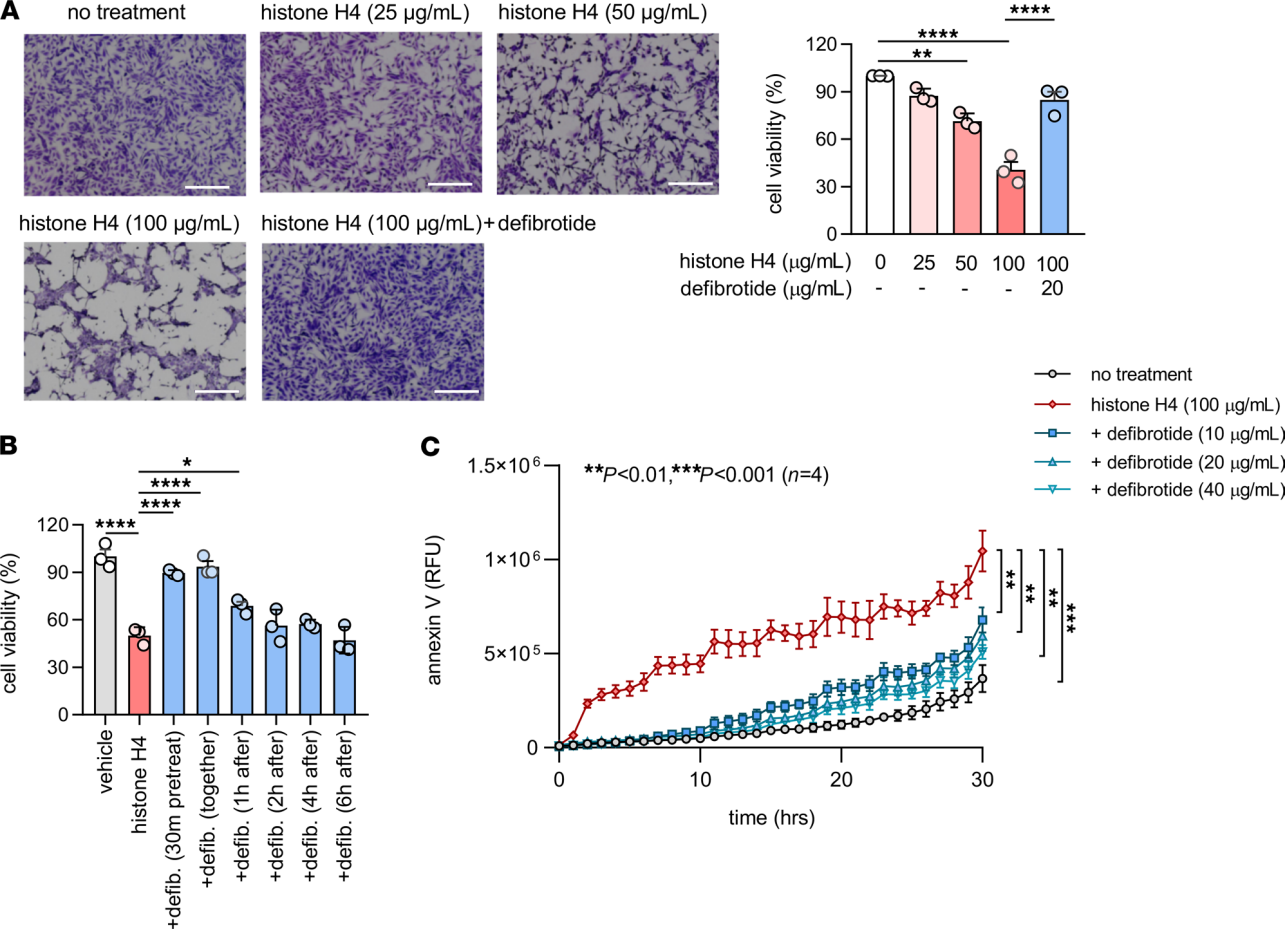
Defibrotide protects HUVECs from histone H4–mediated cell death. (**A**) HUVECs were treated with different doses of histone H4 (0, 25, 50, and 100 μg/mL) in the presence or absence of defibrotide (20 μg/mL). After 24 hours, HUVECs were stained with crystal violet solution for 10 minutes, and absorbance was measured at 570 nm to determine cell viability. Mean ± SD for 3 independent experiments, along with representative images, are presented; **P < 0.01, ***P < 0.001, and ****P < 0.0001 by 1-way ANOVA corrected by Tukey’s multiple comparisons test. Scale bars: 500 μm. (**B**) HUVECs were treated with histone H4 (25 μg/mL) in the presence or absence of defibrotide (20 μg/mL, added at different time points relative to histone H4). After 24 hours, HUVECs were stained with crystal violet solution for 10 minutes, and absorbance was measured at 570 nm to determine cell viability. Mean ± SD is presented for 1 representative experiment out of 3 independent experiments,all with similar results; *P < 0.05 and ****P < 0.0001 by 1-way ANOVA corrected by Tukey’s test. (**C**) HUVECs were treated with histone H4 and different doses of defibrotide in the presence of annexin V red agent. The plate was imaged every hour using the IncuCyte S3 timelapse microscope for 30 hours. Mean ± SD is presented for 1 representative experiment out of 3 independent experiments,all with similar results; **P < 0.01 and ***P < 0.001 by 2-way ANOVA corrected by Dunnett’s test.

**Figure 6:**
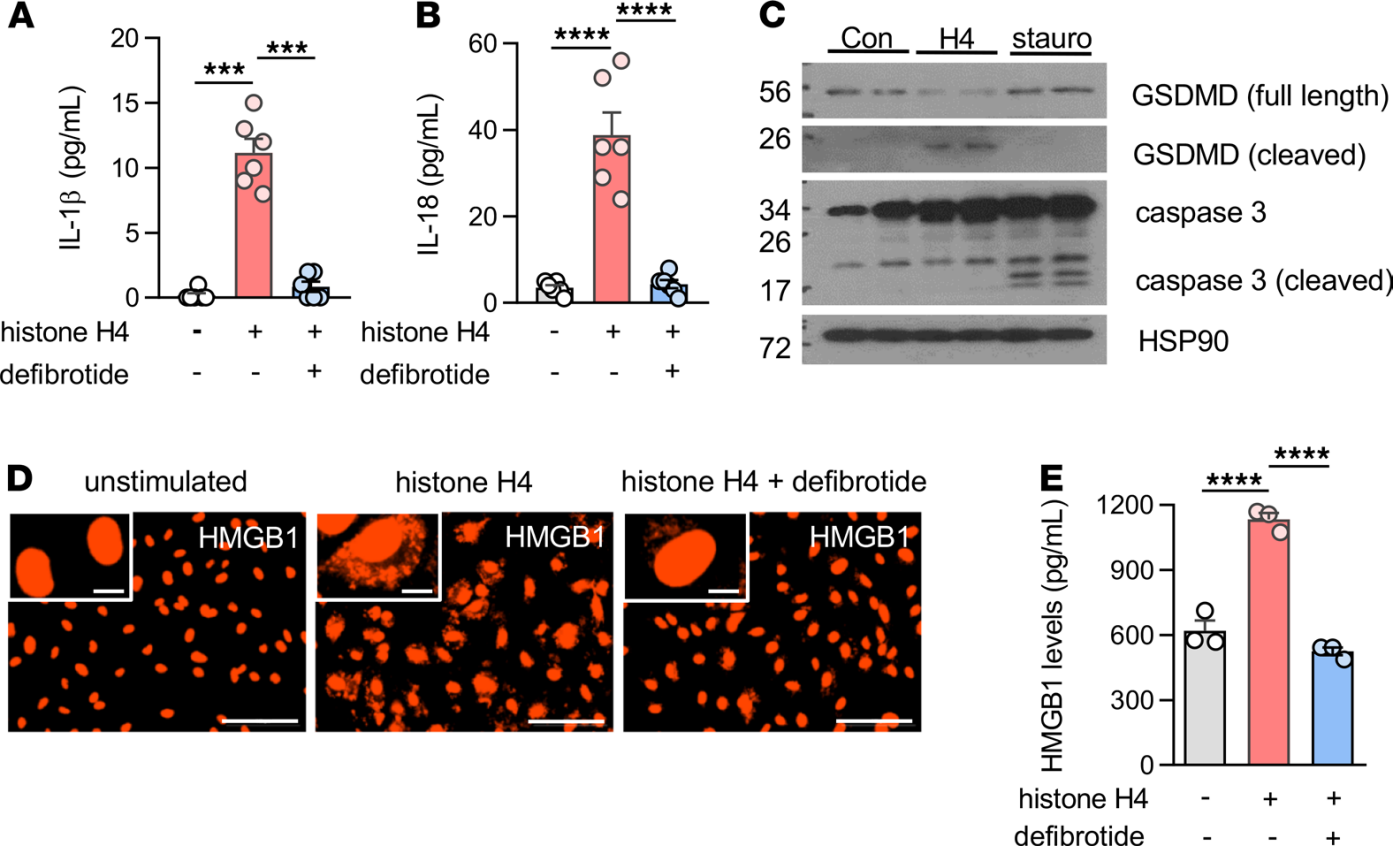
Defibrotide protects HUVECs from histone H4–mediated pyroptosis. (**A** and **B**) HUVECs were treated with histone H4 (100 μg/mL) ± defibrotide (20 μg/mL) for 4 hours. The concentrations of IL-1β (**A**) and IL-18 (**B**) were determined in supernatants (*n =* 6 independent experiments); ***P < 0.001 and ****P < 0.0001 by 1-way ANOVA corrected by Dunnett’s test. Data were presented as mean ± SD. (**C**) Immunoblotting detection of activated gasdermin D (GSDMD) and caspase 3 in cell lysates. HUVECs were treated with histone H4 (100 μg/mL) or staurosporine (50 nM) for 6 hours before collecting the cell lysates. Con, control; H4, histone H4; stauro, staurosporine. (**D** and **E**) HUVECs were treated as in **A** and **B**, and HMGB1 translocation (**D**) and secretion (**E**) were determined by microscopy and supernatant ELISA, respectively (*n =* 3 independent experiments); ****P < 0.0001 by 1-way ANOVA corrected by Dunnett’s test. Scale bars: 100 μm (primary image) and 10 μm (inset). Data were presented as mean ± SD.

The above assays were focused on relatively short cell culture times — typically 6 hours. However, we questioned whether the impact of defibrotide on histone H4–mediated HUVEC activation would persist over longer periods of time. As reported previously, histones go beyond endothelial cell activation and become cytotoxic upon prolonged exposure in culture (17, 22). Indeed, we found remarkable protection of cell viability by defibrotide over a 24-hour period (Figure 5A). We additionally found that 30-minute pretreatment with defibrotide was not absolutely necessary, as adding defibrotide 1 hour (but not later) after histone H4 preserved at least some of the protective effects (Figure 5B). A similar protective effect of defibrotide on cell viability was also observed when HDMVECs were cultured together with histone H4 (Supplemental Figure 9). To further confirm these findings, we varied the experiment by introducing kinetic monitoring of surface phosphatidylserine exposure as measured by annexin V binding. We found a dose-dependent relationship between histone H4 and annexin V binding (Supplemental Figure 10) and found strong and stepwise protection when HUVECs were also cultured with defibrotide concentrations ranging from 10 to 40 μg/mL (Figure 5C). Since previous work has revealed that defibrotide acts as an adenosine receptor agonist in some contexts (46), we asked whether adenosine A_2A_ or A_2B_ receptor antagonists could abolish the protective effects of defibrotide. These adenosine receptors are coupled to Gs proteins that favor intracellular cyclic AMP production. We did not, however, find any role for the A_2A_ antagonist SCH 58261 or the A_2B_ antagonist PSB 603 in negating defibrotide’s protection against annexin V binding (Supplemental Figure 11). We also assessed wortmannin, which has been reported to inhibit defibrotide uptake by endothelial cells (47), but we did not see an effect in our system (Supplemental Figure 11). We did, though, find increased levels of both IL-1β and IL-18 in culture supernatants of HUVECs stimulated by histone H4, both of which were reduced by defibrotide (Figure 6, A and B), supporting the idea that histone H4–mediated cell death may be on the spectrum of pyroptosis. To verify this hypothesis, gasdermin D (GSDMD, a critical protein mediating pyroptosis) and caspase 3 were characterized in HUVEC protein lysates cultured with either histone H4 or an apoptosis inducer, staurosporine. In the histone H4–cultured HUVEC lysates, we found decreased expression of full-length GSDMD but increased expression of cleaved GSDMD, indicating that the type of HUVEC death triggered by histone H4 is pyroptosis but not apoptosis (Figure 6C). To further substantiate these data, we also assessed translocation and subsequent release of the alarmin HMGB1, which is known to track with inflammatory forms of cell death including pyroptosis (48). By microscopy, we observed the translocation of HMGB1 from nucleus to cytoplasm upon exposure of HUVECs to histone H4, with reversal of this effect by defibrotide (Figure 6D). Measurement of HMGB1 in culture supernatants mirrored these findings, with histone H4 triggering HMGB1 release and defibrotide neutralizing that effect (Figure 6E). We also asked whether defibrotide can bind HMGB1, which is, like histone H4, a potentially cytotoxic cationic protein. Consistent with our hypothesis, an EMSA experiment suggested a direct interaction between HMGB1 and defibrotide (Supplemental Figure 12), hinting at another mechanism by which defibrotide might restrain inflammation downstream of histones and NETs. Taken together, these data demonstrate that longer-term exposure of HUVECs to histone H4 triggers pyroptosis and that defibrotide’s antihistone effects are sustained in culture for up to 24 hours.

### Defibrotide counters histone-accelerated venous thrombosis in mice.

**Figure 7:**
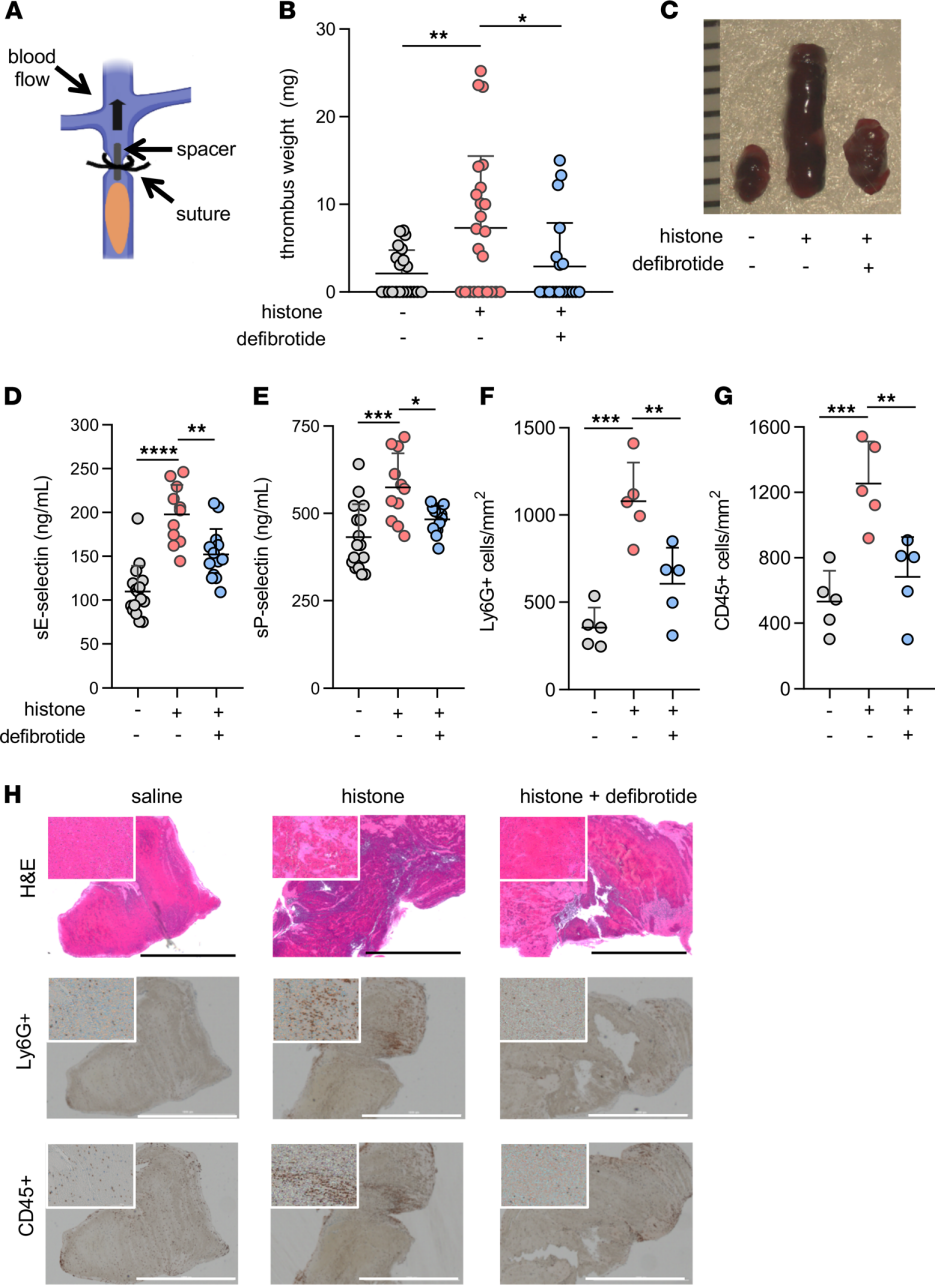
Defibrotide alleviates histone-mediated endothelial activation and venous thrombosis in mice. (**A**) Thrombus initiation in the IVC via placement of a fixed suture over a spacer that was subsequently removed. (**B**) Mice were injected with either histone (10 mg/kg) or saline via tail vein 1 hour prior to surgery. Meanwhile, defibrotide (150 mg/kg) or saline was administered by retro-orbital injection 24 hours prior to surgery and then immediately following closure of the abdomen. Thrombus weight was determined 24 hours later. Scatter plots are presented, with each data point representing a unique mouse (horizontal bars represent mean + SD); *P < 0.05 and **P < 0.01 by Kruskal-Wallis test followed by Dunn’s multiple comparison test. Data were presented as mean ± SD. (**C**) Representative thrombi from the experiments presented in panel **B** with rulers measuring thrombi in millimeters. (**D** and **E**) Serum samples from the experiments presented in **B** were tested for soluble E-selectin (**D**) and soluble P-selectin (**E**) by ELISA; *P < 0.05, **P < 0.01, ***P < 0.001, and ****P < 0.0001 by 1-way ANOVA corrected by Dunn’s multiple comparison test. Data were presented as mean ± SD. (**F**–**H**) Thrombus sections from **B** were stained for Ly6G^+^ and CD45^+^ cells. Positively stained cells were quantified in 4 randomly selected fields for each thrombus. **P < 0.01 and ***P < 0.001 by 1-way ANOVA corrected by Dunn’s multiple comparison test. Scale bars: 1000 μm. Data is presented as mean ± SD.

To determine the potential in vivo relevance of these findings, we employed a model of venous thrombosis in which a constricting ligature is fixed around the inferior vena cava (IVC); then, the presence and potential characteristics of thrombosis were assessed 24 hours later (Figure 7A; refs. 6, 49). First, we asked whether thrombus accretion was impacted by injection of calf thymus histones, and we indeed found this to be the case (Figure 7, B and C); at the same time, extensive review of kidney sections did not reveal spontaneous structures in glomeruli or vessels suggestive of thrombi (demonstrating that a second hit was needed in addition to histone injection). We also did not find that histone injection boosted the levels of myeloperoxidase-DNA complexes (NET remnants) in blood (data not shown). As part of these experiments, we also administered defibrotide i.v. shortly after injection of the histones. With this approach, both thrombus accretion (Figure 7, B and C) and thrombus length (Supplemental Figure 13) were reduced essentially to the levels seen in control mice. In support of endothelial cell activation contributing to the histone-accentuated thrombosis phenotype, both soluble E-selectin and soluble P-selectin tracked closely with thrombus accretion (Figure 7, D and E), as did infiltration of leukocytes, whether scored as Ly6G^+^ (neutrophils) or CD45^+^ (most leukocytes; Figure 7, F–H). In support of this concept, there was a strong correlation between either soluble E-selectin or soluble P-selectin and thrombus size (Supplemental Figure 14). Taken together, these data confirm the proinflammatory and prothrombotic impact of histones in vivo and demonstrate that defibrotide has the potential to neutralize these properties.

## Discussion

As evidence continues to implicate NETs and NET-derived histones in the pathophysiology of disease states ranging from infection (including COVID-19) to autoimmunity to cancer (50), the search for NET-targeting therapeutics takes on additional importance. Here, we explored the extent to which an US Food and Drug Administration–approved drug defibrotide might protect endothelial cells from NETs and extracellular histones. We found defibrotide to counteract endothelial cell activation and hypercoagulability triggered by NETs and histone H4. Mechanistically, our evidence points to a direct interaction between defibrotide and cationic proteins, such as histone H4, as an important aspect of these protective effects.

Polyanionic substances naturally exist in the extracellular environment where they play a variety of biological roles (51). Unfractionated heparin and suramin are examples of how polyanionic drugs may be leveraged clinically. These agents can potentially bind cationic microbe-derived proteins, as well as cationic tumor cytokines and receptors, in the treatment of infectious diseases and cancer, respectively (52–56). In previous work, defibrotide’s polyanionic properties have been shown to include binding with high affinity to specific heparin-binding proteins including basic fibroblast growth factor (bFGF; ref. 40). Interestingly, defibrotide (oligonucleotides) and heparins (proteoglycans) share similarities in charge distributions and binding patterns (57).

Histones bind DNA tightly mainly due to charge-charge interactions, with a possible role for specific DNA sequence motifs (58). Our results found a strong interaction between histone H4 and defibrotide, which was very resistant to dissociation. Considering defibrotide is a natural product (i.e., not produced by a DNA synthesizer), the possibility of any specific sequence dominating its effect is low. We therefore speculate that the main binding force between histone H4 and defibrotide also comes from charge-charge interactions. An interesting unknown is the extent to which the degradation of defibrotide in vivo may be delayed upon binding histone H4. Typically, phosphodiester oligonucleotides would be rapidly degraded in plasma; however, we found a protective role in an animal model over 24 hours without the need for redosing. A deeper understanding of these in vivo properties should be a priority for future research.

As a major component of NETs, histones are one factor that contributes to vascular dysfunction during sepsis, where they trigger neutrophil migration, endothelial injury, hemorrhage, and thrombosis (17). Compared with other histones, histone H4 has the strongest impact on platelets, enhancing thrombin generation and accelerating thrombosis (59). Histone H4 has also been reported as the major histone mediator of membrane lysis of smooth muscle cells, as well as arterial tissue damage and inflammation in atherosclerosis (22). Our study has now also revealed that neutralizing histone H4 significantly mitigates NET-mediated activation of HUVECs. Whether defibrotide preferentially neutralizes histone H4 as compared with other histones is an area for further research.

Multiple organ dysfunction syndrome (MODS) is widely considered to be the leading cause of morbidity and mortality for patients admitted to an intensive care unit, where it encompasses heterogeneous disease states such as sepsis, shock, trauma, severe burn, and pancreatitis (60–63). Systemic inflammation and vascular coagulopathy account for the main pathological processes of MODS (64) — and they, of course, also characterize aspects of COVID-19 (65–68) and the closely related catastrophic antiphospholipid syndrome (69). In MODS, endothelial cell activation is considered a precursor to tissue damage and end-organ dysfunction, with upregulation of adhesion molecules triggered by cytokines, microbial proteins, and various cationic proteins from necrotic cells (70). One recent study evaluated circulating histones in a cohort of 420 ICU patients with sepsis, severe trauma, or severe pancreatitis and identified circulating histones as major mediators of MODS in these patients (71). An important future direction of this work will be to characterize the role of histones and defibrotide in the context of in vivo models that interrogate the microvasculature, where much of the pathology of MODS resides. One may then be able to consider whether administration of defibrotide in an early phase of MODS might neutralize cationic proteins such as histones to stabilize the endothelium and break the vicious thromboinflammatory cycle. Indeed, a number of clinical trials focused on defibrotide therapy for COVID-19 are currently underway or recently completed (NCT04530604, NCT04335201, NCT04348383, NCT04652115; ClinicalTrials.gov). These, and potentially other future trials, should help elucidate the extent to which defibrotide and other histone-neutralizing agents may have a role in combatting NET-mediated disease states.

## Methods

### Cell culture and reagents.

HUVECs and HDMVECs purchased from ATCC were cultured in EBM supplemented with EGM-2MV singleQuots (Lonza) without hydrocortisone in 0.2% gelatin-coated tissue culture plates. All experiments were performed using HUVECs of passage 6 or lower. Recombinant histone H4 was purchased from Cayman (catalog 10264) for in vitro experiments. Histone from calf thymus was purchased from MilliporeSigma (catalog 10223565001). Anti–histone H4 was from Cell Signaling Technology (catalog 2592). TLR2 inhibitor C29 was from Medchemexpress (catalog HY-100461), and TLR4 inhibitor TAK 242 was from MilliporeSigma (catalog 614316). Citrullinated histone H4 (catalog 17927), SCH 58261 (catalog 19676), PSB 603 (catalog 25637), and wortmannin (catalog 10010591) were purchased from Cayman.

### NET isolation.

Neutrophils were isolated from healthy volunteers. NETs were stimulated with 500 nM PMA or 10 μM calcium ionophore A23187 and purified as described previously (72).

### Quantitative PCR (qPCR).

Total RNA was isolated using Direct-zol RNA MiniPrep kit (Zymo Research) according to manufacturer’s instructions. In total, 200 ng of RNA from each sample was reverse transcribed using random hexamer primed single-strand cDNA (10 minutes at 25°C, 15 minutes at 42°C, 5 minutes at 99°C) by MMLV Reverse Transcriptase (Invitrogen). cDNA was amplified using Fast SYBR Green Mastermix (Invitrogen) on a ViiA7-Realtime qPCR System (Invitrogen). Expression level of mRNAs were normalized to β-actin. All gene primers were purchased from Qiagen.

### Neutrophil adhesion assay.

Monolayer HUVECs were cultured with or without NETs for 4 hours. Isolated fresh neutrophils were labeled with calcein-AM (C1430, Thermo Fisher Scientific) for 30 minutes at 37°C, and then 6 × 10^5^ neutrophils per well were added to the washed (RPMI + 3% BSA) monolayer for 20 minutes. After washing with prewarmed HBSS, adherent neutrophil fluorescence was measured with a Cytation 5 Cell Imaging Multi-Mode Reader (BioTek) at 485 and 535 nm (excitation and emission wavelengths, respectively).

### TF activity.

Cell lysates were prepared with 150 μL 15 mM octyl-β-D-glycopyranoside (MilliporeSigma) for 15 minutes at 37°C. TF activity was measured using TF Human Chromogenic Activity Assay Kit (ab108906, Abcam) according to the manufacturer’s instructions.

### Permeability assay.

Permeability was assessed by measuring the passage of horseradish peroxidase (HRP) through endothelial cell monolayers in a Transwell system (Cell Biologics). Briefly, HUVECs were plated at 50,000 cells/mL in the Transwells and allowed to grow to confluence; they were then cultured in EBM-2 media with 1% FBS in the upper and lower chambers. Treatments, including NETs (1 μg DNA content/mL) and/or defibrotide (10 μg/mL), were added to the upper chambers along with HRP, and aliquots of the media in the lower chambers were collected at various time points. The amount of HRP was quantified by the addition of 3,3′,5,5′-tetramethylbenzidine benzidine followed by 2N sulfuric acid stop solution. Absorbance was read at 450 nm in a plate reader.

### In-cell ELISA.

Confluent monolayers of HUVECs in 96-well microplates were incubated with NETs for 6 hours. Some cultures were additionally supplemented with defibrotide. Cells were fixed by adding an equal volume of 8% paraformaldehyde for 30 minutes. Blocking was with 2× blocking solution (ab111541, Abcam) at room temperature for 2 hours. After washing with PBS, cells were incubated with 5 μg/mL primary mouse anti-human antibodies against E-selectin (BBA26, R&D), VCAM-1 (BBA5, R&D), or ICAM-1 (ab2213, Abcam) at 4°C overnight. Next, 100 μL of diluted HRP conjugated rabbit anti–mouse IgG (1:2000, Jackson ImmunoResearch, catalog 315-035-003) in 1× blocking solution was added and incubated at room temperature for 1 hour. After washing thoroughly with PBS, 100 μL of TMB substrate was added, and blue color development was measured at OD 650 nm with a Cytation 5 Cell Imaging Multi-Mode Reader (BioTek). The signals were corrected by subtracting the mean signal of wells incubated in the absence of the primary antibody.

### RNA-seq.

Total RNA from cells was isolated using RNeasy Plus Mini Kit (74134, Qiagen) according to manufacturer’s instructions. Sequencing was performed by the UM Advanced Genomics Core, with libraries constructed and subsequently subjected to 150 paired-end cycles on the NovaSeq-6000 platform (Illumina). FastQC (v0.11.8) was used to ensure the quality of data, and adapter sequences were trimmed from raw reads using Cutadapt (v2.3) prior to alignment. Reads were mapped to the reference genome GRCh38 (ENSEMBL) using STAR (v2.6.1b) and assigned count estimates to genes with RSEM (v1.3.1). Alignment options followed ENCODE standards for RNA-seq. FastQC was used in an additional postalignment step to ensure that only high-quality data were used for expression quantitation and differential expression. Differential expression data were prefiltered to remove genes with 0 counts in all samples. Differential gene expression analysis was performed using DESeq2, using a negative binomial generalized linear model (thresholds: linear fold change >1 .5 or < –1.5, Benjamini-Hochberg FDR *P*_adj_ < 0.05). Plots were generated using variations of DESeq2 plotting functions and other packages with R version 3.3.3. Functional analysis, including candidate pathways activated or inhibited in comparisons and GO-term enrichments, was performed using iPathway Guide (Advaita). RNA-seq data discussed in this publication have been deposited in NCBI’s Gene Expression Omnibus (GEO GSE179828; https://www.ncbi.nlm.nih.gov/geo/query/acc.cgi?acc=GSE179828).

### Quantification of cytokines.

Cytokines were quantified in supernatants using human IL-1β DuoSet ELISA kit (DY201, R&D systems), human IL-18 ELISA Kit (7620, MBL International), human IL-8 DuoSet ELISA kit (DY208, R&D systems), and human MCP-1 DuoSet ELISA kit (DY279, R&D systems), according to the manufacturers’ instructions.

### EMSA.

In total, 10 μg of defibrotide was incubated with various concentrations (40 μM, 80 μM, 120 μM) of histone H4 in serum-free RPMI for 1 hour at 37°C to form complexes. Furthermore, 120 μM BSA was used as a negative protein control. Complexes were then run on a 0.5% agarose gel stained with SYBR safe (Invitrogen) for 30 minutes. The gel was imaged on a Typhoon FLA 7000 biomolecular imager (GE Healthcare).

### SPR assay.

SPR studies were performed using the Biacore T200 with His-tagged histone H4 coupled to a NiNTA chip. Defibrotide in a series of concentrations from 0.39 μg/mL to 12.5 μg/mL was injected over the sensor chip at room temperature, using a running buffer of 50 mM HEPES-NaOH (pH 7.4), 100 mM NaCl, and 0.002% surfactant P-20. Resonance was corrected for background using a reference cell without histone H4, and curves were blank subtracted using data acquired with running buffer only. Data were analyzed using BIA Evaluation software (GE Healthcare) to determine binding affinity at steady state. Data shown are representative of 3 independent experiments.

### Crystal violet viability staining.

Cell viability was tested by crystal violet staining as reported previously (73).

### Annexin V staining.

Cells were seeded into a 96-well plate and allowed to adhere overnight. Annexin V reagent (Incucyte Annexin V Green Reagent for Apoptosis; Essen Bioscience, final dilution of 1:200) was added together with histone H4 with or without defibrotide on the following day. Annexin V staining was monitored with the IncuCyte S3 microscopy system every 1 hour for 30 hours. Excitation and emission wavelengths were 490 nm and 515 nm, respectively. Images were collected by a Nikon 20× objective. IncuCyte S3 integrated software (Essen Bioscience) was used to minimize background fluorescence and quantify fluorescent objects.

### Immunoblotting analysis.

Cells were harvested and homogenized with lysis buffer containing 2% SDS, 50 mM Tris-HCl (pH 6.8), 10 mM DTT, 10% glycerol, 0.002% bromphenol blue, and freshly added protease inhibitors. Immunoblotting experiments were performed using specific antibodies. Antibodies used in this study were against caspase 3 (catalog 9662, Cell Signaling Technology), full-length GSDMD (catalog 96458, Cell Signaling Technology), cleaved N-terminal GSDMD (catalog ab215203, Abcam), and Hsp90 (catalog sc-7947, Santa Cruz Biotechnology Inc.).

### Detection of HMGB1.

For immunofluorescence microscopy, 1 × 10^5^ HUVECs/well were seeded onto coverslips coated with 0.2% gelatin the day before experiments. The HUVECs were treated with 100 μg/mL histone H4 in the presence or absence of defibrotide for 24 hours. Cells were fixed with 1% paraformaldehyde for 10 minutes, permeabilized with 0.5% TritonX-100 for 10 minutes, and blocked with 5% FBS for 30 minutes. Then, the cells were intracellularly stained with 5 μg/mL anti-HMGB1 Alexa Fluor 594 (clone 3E8, BioLegend) in blocking buffer overnight at 4°C. Images were collected with a Cytation 5 Cell Imaging Multi-Mode Reader (BioTek). HMGB1 was quantified in supernatants using the HMGB1 ELISA Kit (NBP2-62766, Novus) according to the manufacturer’s instructions.

### Mouse models of venous thrombosis.

Male C57BL/6 mice were purchased from The Jackson Laboratory (stock no. 000664) and used at approximately 10 weeks of age. Large-vein thrombosis was modeled as we have described previously (6). Mice were injected with either histone (10 mg/kg) or saline 1 hour prior to surgery via tail vein. Defibrotide (150 mg/kg) or an equal volume of saline were administered i.v. via retro-orbital injection. The first dosage of defibrotide was given 24 hours prior to surgery, and the second dose was delivered just after closure of the abdomen. Thrombus was determined 24 hours later.

### Quantification of mouse soluble E-selectin and P-selectin.

Soluble E-selectin and P-selectin were quantified in mice sera using the mouse E-selectin Duoset ELISA (DY575, R&D system) and mouse P-selectin Duoset ELISA (DY737, R&D system) according to the manufacturer’s instructions.

### Statistics.

Data analysis was performed with GraphPad Prism software version 8. For continuous variables, group means were compared by 1-way ANOVA (more than 2 groups); correction for multiple comparisons was by Dunnett’s, Sidak’s, or Tukey’s method. For 2 independent variables, group means were compared by 2-way ANOVA (more than 2 groups); correction for multiple comparisons was by Dunnett’s method. Correlations were tested by Pearson’s correlation coefficient. Statistical significance was defined as *P <* 0.05.

### Study approval.

Neutrophils were isolated from healthy volunteers recruited through an IRB-approved advertisement (HUM00044257). All mouse experiments were approved by the University of Michigan IACUC.

## Author contributions

HS, AAG, SAS, QYW, DC, SY, RAA, CL, GS, and PST conducted experiments and analyzed data. HS, YZ, YK, EAF, JDL, JHM, and JSK conceived the study and analyzed data. All authors participated in writing the manuscript and gave approval before submission.

## Supplementary Material

Supplemental data

## Version 1 07/15/2021

In-Press Preview

## Version 2 09/08/2021

Electronic publication
