# Supplementary material for: Endothelium-protective, histone-neutralizing properties of the polyanionic agent defibrotide
Source: JCI Insight. 2021 Sep 8;6(17):e149149. doi: 10.1172/jci.insight.149149 (PMC8492316; doi:10.1172/jci.insight.149149)
Supplement: Supplemental data [file jciinsight-6-149149-s008.pdf]

Supplementary Materials

Endothelium-protective histone-neutralizing properties of the polyanionic agent defibrotide

Shi *et al.*

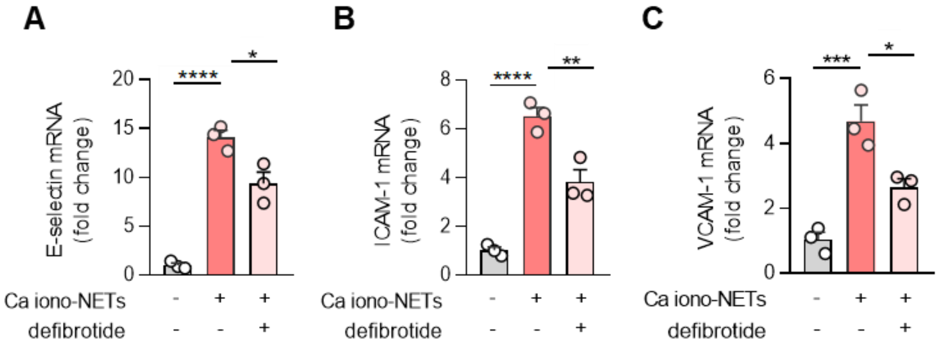

Supplementary Figure 1: Defibrotide inhibits the activation of cultured HUVECs by calcium ionophore-induced NETs. A-C, HUVECs were pretreated with defibrotide (10 μg/ml) for 30 minutes, followed by isolated NETs (1 μg DNA content/ml triggered by 10 μM calcium ionophore) for 4 hours. E-selectin (A), ICAM-1 (B), and VCAM-1 (C) mRNA levels were determined by qPCR. Mean ± standard deviation is presented for one representative experiment out of three independent experiments, all with similar results; ****$p<0.0001$ by one-way ANOVA corrected by Dunnett's test.

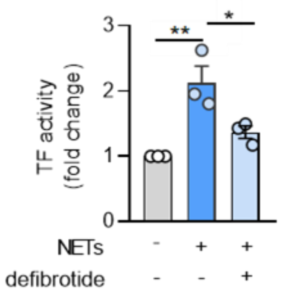

Supplementary Figure 2: Defibrotide inhibits the tissue factor activity of cultured HUVECs by NETs. HUVEC lysates were prepared and tissue factor activity was determined as described in Methods. Mean ± standard deviations are presented for n=3 independent experiments; *p<0.05 and **p<0.01 by one-way ANOVA corrected by Dunnett’s test.

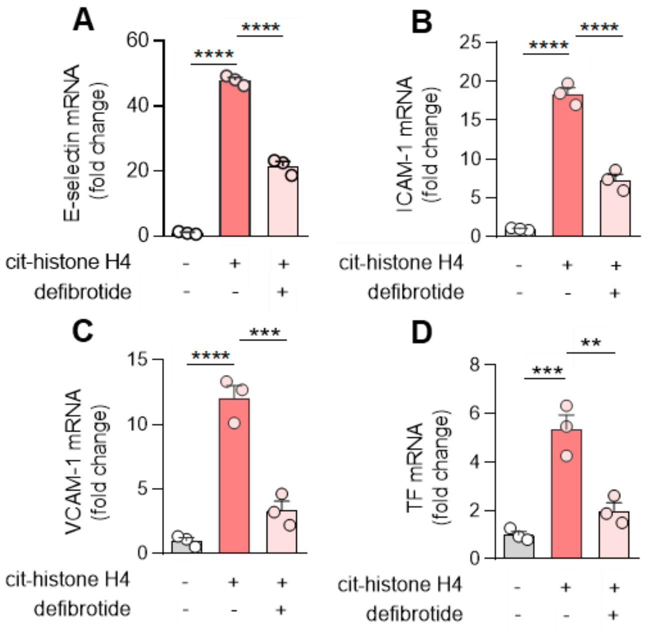

Supplementary Figure 3: Defibrotide mitigates HUVEC activation by citrullinated histone H4 (cit-histone H4). A-D, HUVECs were pretreated with defibrotide (10 μg/ml) for 30 minutes, followed by recombinant citrullinated histone H4 (25 μg/ml) for 4 hours. E-selectin (A), ICAM-1 (B), VCAM-1 (C), and tissue factor (D) mRNA levels were determined by qPCR. Mean ± standard deviation is presented for one representative experiment out of three independent experiments, all with similar results; **$p < 0.01$, ***$p < 0.001$, ****$p < 0.0001$ by one-way ANOVA corrected by Dunnett's test.

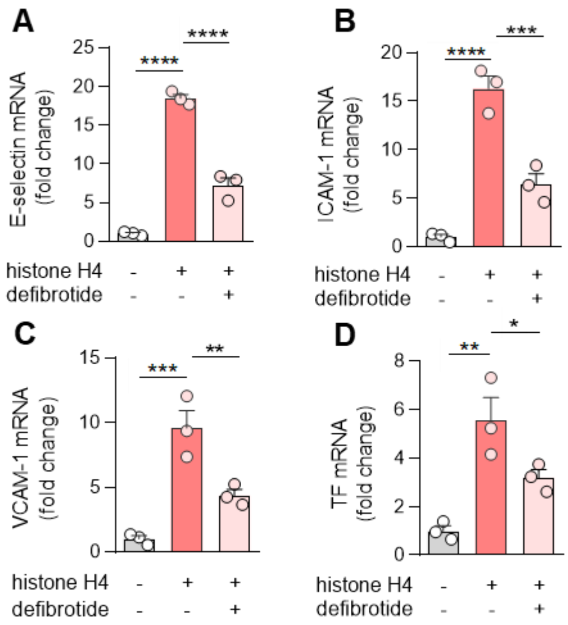

Supplementary Figure 4: Defibrotide attenuates human dermal microvascular endothelial cell (HMVEC) activation by extracellular histone H4. **A-D,** HMVECs were pretreated with defibrotide (10 µg/ml) for 30 minutes, followed by recombinant histone H4 (25 µg/ml) for 4 hours. E-selectin (A), ICAM-1 (B), VCAM-1 (C), and tissue factor (D) mRNA levels were determined by qPCR. Mean ± standard deviation is presented for one representative experiment out of three independent experiments, all with similar results; *$p < 0.05$, **$p < 0.01$, ***$p < 0.001$, ****$p < 0.0001$ by one-way ANOVA corrected by Dunnett's test.

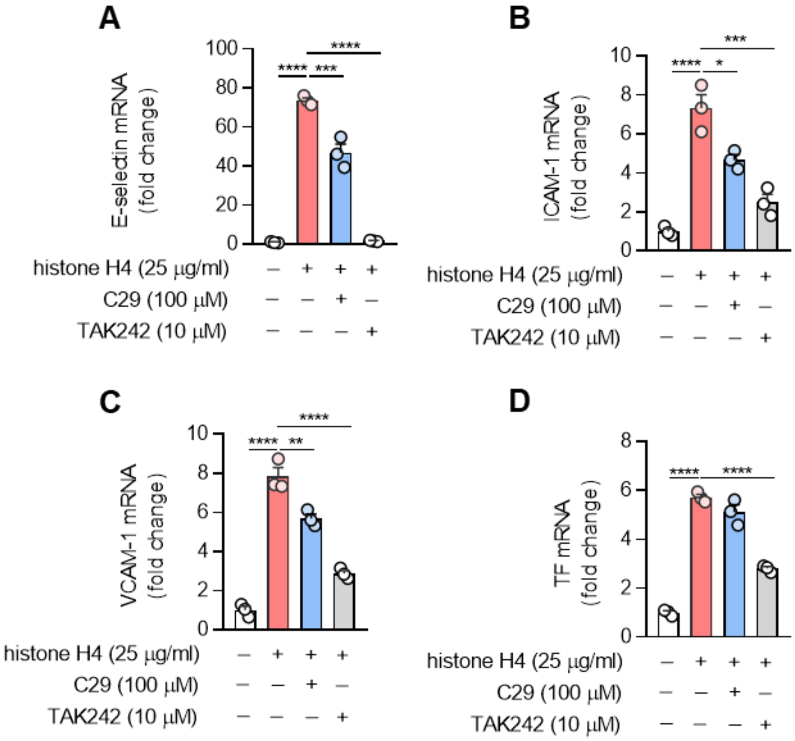

Supplementary Figure 5: Toll-like receptor inhibitors mitigate the activation of cultured HUVECs by histone H4. A-D, HUVECs were pretreated with C29 (TLR2 inhibitor) or TAK242 (TLR4 inhibitor) for 1 hour, followed by the addition of histone H4 (25 µg/ml) for 4 hours. E-selectin (A), ICAM-1 (B), VCAM-1 (C) and tissue factor (D) mRNA levels were determined by quantitative PCR. Mean ± standard deviation is presented for one representative experiment out of three independent experiments, all with similar results; *p<0.05, **p<0.01, ***p<0.001, and ****p<0.0001 by one-way ANOVA corrected by Dunnett's test.

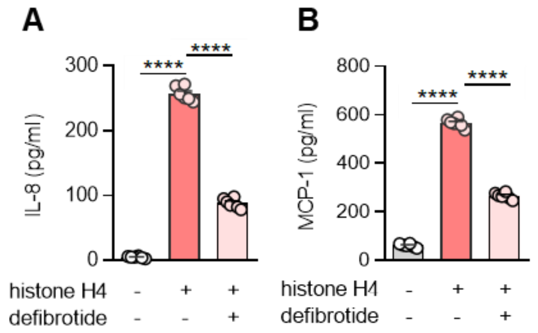

Supplementary Figure 6: Defibrotide protects HUVECs from histone H4-mediated chemokine secretion. HUVECs were treated with histone H4 (25 µg/ml) ± defibrotide (10 µg/ml) for 4 hours. The concentrations of IL-8 (A) and MCP-1 (B) were determined in supernatants (n=6 independent experiments); ****p<0.0001 by one-way ANOVA corrected by Dunnett's test.

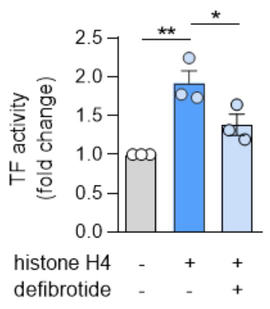

Supplementary Figure 7: Defibrotide inhibits the tissue factor activity of cultured HUVECs by histone H4. HUVEC lysates were prepared and tissue factor activity was determined as described in Methods. Mean ± standard deviations are presented for n=3 independent experiments; *p<0.05 and **p<0.01 by one-way ANOVA corrected by Dunnett's test.

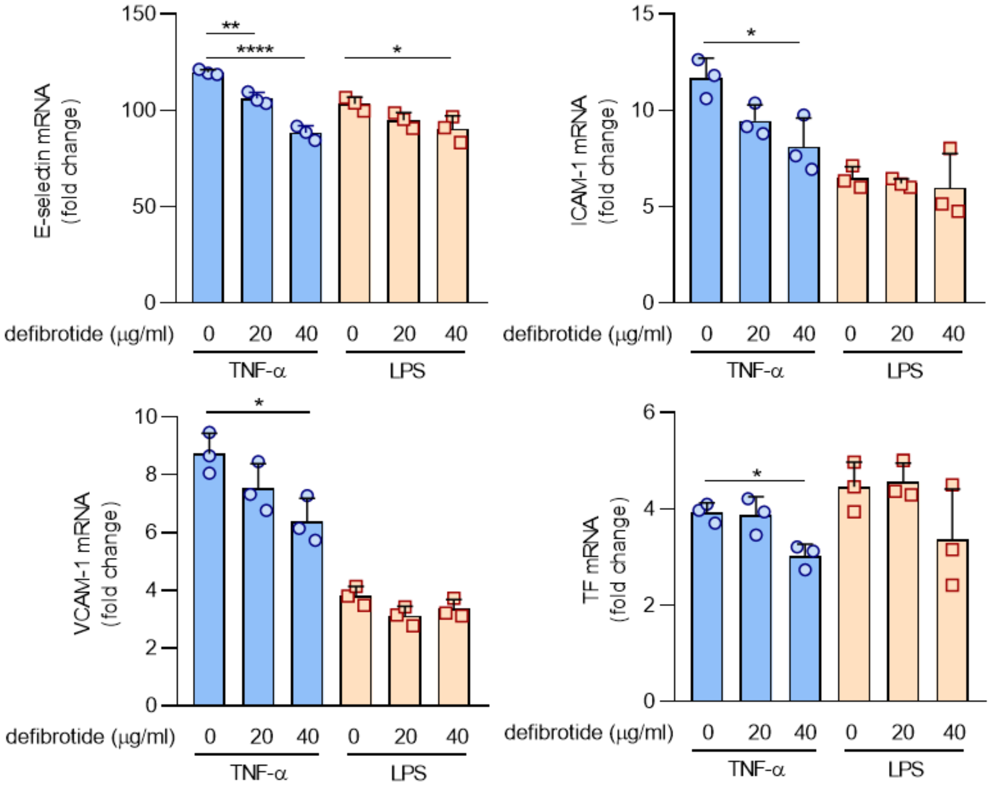

Supplementary Figure 8: Defibrotide shows mild protection when HUVECs are activated by TNF-α and little to no protection when HUVECs are activated by lipopolysaccharide (LPS). A-D, HUVECs were pretreated with defibrotide (20 or 40 μg/ml) for 30 minutes, followed by TNF-α (20 nM) or LPS (1 μg/ml) for 4 hours. E-selectin (A), ICAM-1 (B), VCAM-1 (C), and tissue factor (D) mRNA levels were determined by qPCR. Mean ± standard deviation is presented for one representative experiment out of three independent experiments, all with similar results. *$p<0.05$, **$p < 0.01$, ****$p < 0.0001$ by one-way ANOVA corrected by Dunnett's test.

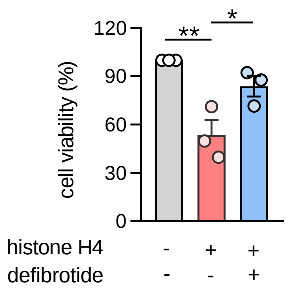

Supplementary Figure 9: Defibrotide protects HMVECs from histone H4-mediated cell death. HMVECs were treated with histone H4 (25 µg/ml) in the presence or absence of defibrotide (20 µg/ml). After 24 hours, HVECs were stained with crystal violet solution for 10 minutes, and absorbance was measured at 570 nm to determine cell viability. Mean ± standard deviation for three independent experiments is presented; *p<0.05 and **p<0.01 by one-way ANOVA corrected by Dunnett's test**.**

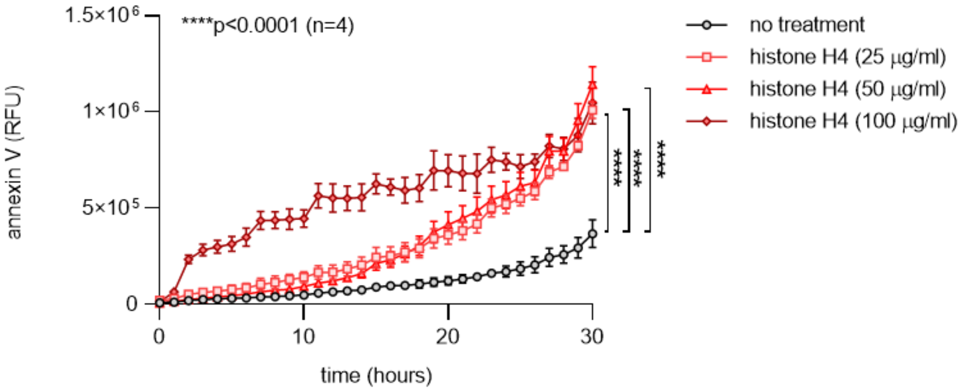

Supplementary Figure 10: HUVECs expose phosphatidylserine in response to histone H4. HUVECs were treated with different concentrations of histone H4 in the presence of Annexin V red agent. The plate was imaged every hour using the IncuCyte® S3 timelapse microscope for 30 hours. Mean ± standard deviation for three independent experiments is presented; ****p<0.0001by two-way ANOVA corrected by Dunnett's test.

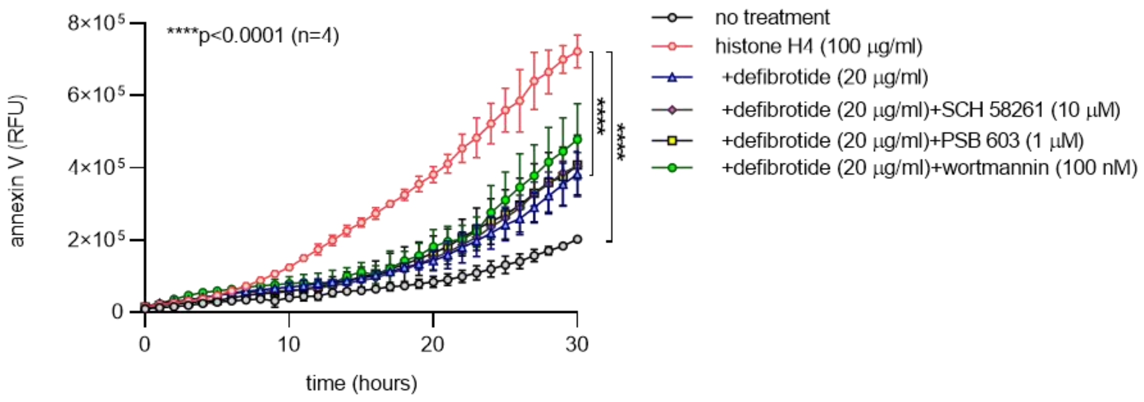

Supplementary Figure 11: Adenosine receptor antagonists and wortmannin do not abolish the protective effect of defibrotide on HUVECs exposing phosphatidylserine in response to histone H4. HUVECs were treated with histone H4 and defibrotide as indicated. Some samples were additionally treated with the adenosine A_{2A} receptor antagonist SCH 58261, the adenosine A_{2B} receptor antagonist PSB 603, or wortmannin in the presence of Annexin V red agent. The plate was imaged every hour using the IncuCyte® S3 timelapse microscope for 30 hours. Mean ± standard deviation for three independent experiments is presented; ****p<0.0001by two-way ANOVA corrected by Dunnett’s test.

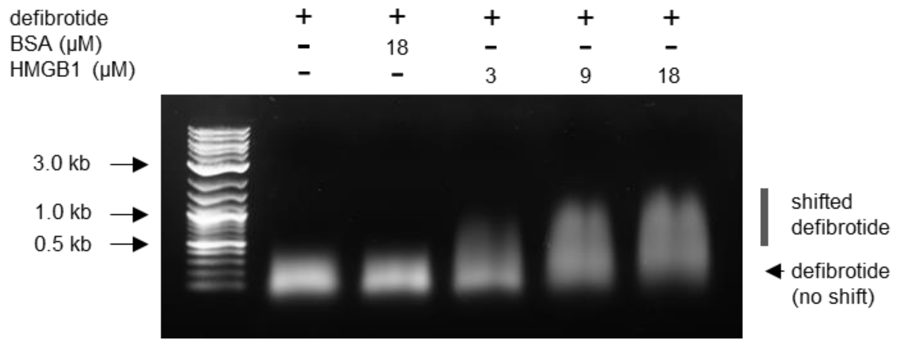

Supplementary Figure 12: Direct interaction between HMGB1 and defibrotide revealed by electrophoretic mobility shift assay. Defibrotide and HMGB1 were incubated at 37°C for 30 minutes and then resolved on a 0.5% agarose gel.

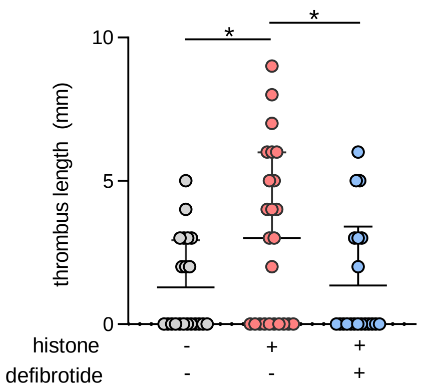

Supplementary Figure 13: Defibrotide reduces the length of histone-mediated venous thrombi. Mice were injected with either histone (10 mg/kg) or saline via tail vein 1 hour prior to surgery via tail. Meanwhile, defibrotide (150 mg/kg) or saline was administered by retro-orbital injection 24 hours prior to surgery and then immediately following closure of the abdomen. Thrombus length was determined 24 hours later. Scatter plots are presented with each data point representing a unique mouse (horizontal bars=means; *p<0.05 by one-way ANOVA corrected by Dunnett's test. Data is presented by mean ± standard deviation.

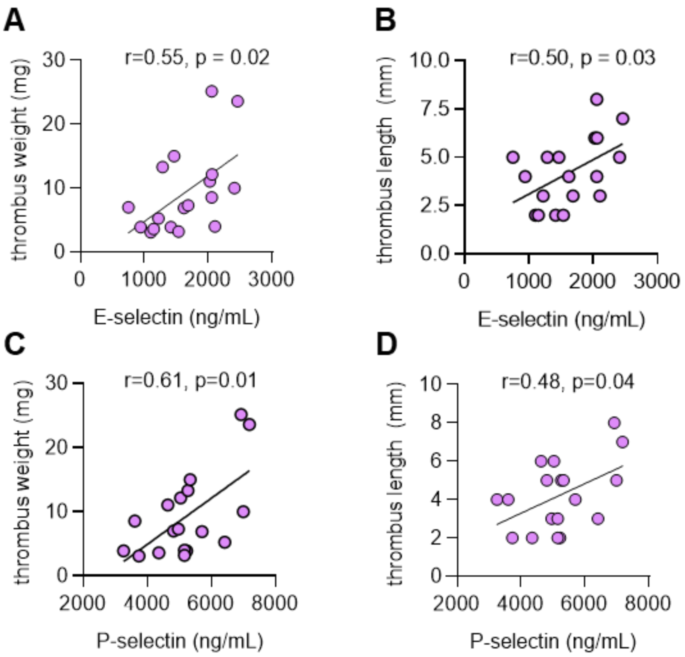

Supplementary Figure 14: Association between soluble E-selectin or soluble P-selectin and thrombus size in mice. A-D, the data presented in Figure 7 and Supplementary Figure 13 are presented here as scatter plots. Correlations were tested by Pearson's method.
